# Good to Excellent Functional Short-Term Outcome and Low Revision Rates Following Primary Anterior Cruciate Ligament Repair Using Suture Augmentation

**DOI:** 10.3390/jcm9103068

**Published:** 2020-09-23

**Authors:** Kristian Nikolaus Schneider, Benedikt Schliemann, Georg Gosheger, Christoph Theil, Jan Weller, Pranai K Buddhdev, Georg Ahlbäumer

**Affiliations:** 1 Department of Orthopedics and Trauma Surgery, Klinik Gut, St. Moritz, 7500 St. Moritz, Switzerland; kristian.schneider@ukmuenster.de (K.N.S.); janfredericweller@gmail.com (J.W.); 2 Department of Orthopedics and Tumor Orthopedics, University Hospital of Münster, 48149 Münster, Germany; georg.gosheger@ukmuenster.de (G.G.); christoph.theil@ukmuenster.de (C.T.); 3 Department of Trauma-, Hand- and Reconstructive Surgery, University Hospital of Münster, 48149 Münster, Germany; benedikt.schliemann@ukmuenster.de; 4 Department of Trauma Surgery, Broomfield Hospital Essex, Chelmsford CM1 7ET, UK; pranai2000@hotmail.com

**Keywords:** anterior cruciate ligament, ACL, injury, tear, rupture, repair, suture augmentation, internal brace

## Abstract

The aim of this study was to evaluate the functional outcome of primary anterior cruciate ligament (ACL) repair using suture augmentation (SA) in 93 consecutive patients (67 female) with a minimum follow-up of 12 months. Patients’ outcomes were determined using International Knee Documentation Committee (IKDC) score, Lysholm score (LS) and Tegner score (TS). Knee-laxity was assessed using the KT-1000 arthrometer. Eighty-eight patients (67 female, mean age 42 years ± standard deviation (SD) 13) were available for follow-up after a mean time of 21 months (range 12–39). Three patients (3%) underwent revision surgery and were excluded from functional analysis. The mean IKDC score was 87.4 ± 11, mean LS was 92.6 ± 11, mean pre-traumatic TS was 6 ± 2 and mean postoperative TS was 6 ± 2, with a mean difference (TS_Diff_) of 1 ± 1. The interval from injury to surgery had no significant impact on the postoperative IKDC (*p* = 0.228), LS (*p* = 0.377) and TS_Diff_ (*p* = 0.572). Patients’ age (>40 years), BMI (>30) and coexisting ligament or meniscal injuries did not seem to influence postoperative functional results. Primary ACL repair using SA provides good to excellent functional outcomes with a low probability of revision surgery at a minimum of 12 months.

## 1. Introduction

The optimal treatment of an anterior cruciate ligament (ACL) tear remains debatable; however, in the demanding athletic patient, ACL reconstruction using a tendon autograft is usually recommended [1,2,3]. Contrary to this, in the 1970s and 1980s, open primary ACL repair was the gold standard surgical treatment [4,5,6]. Due to the poor biological healing capacity of the ACL with synovial fluid creating “a hostile environment” and intra-articular movements preventing the required formation of a stable fibrin-platelet scaffold, high re-rupture rates and simultaneous advancements in ACL reconstruction led to an almost complete abandonment of open ACL repair in the 1990s [4,5,7]. A resurgence of focused attention on restoring native anatomy, preservation of proprioceptive abilities and reduction in donor site morbidity have led to a renewed interest in ACL repair for patients presenting with a proximal tear pattern, with excellent tissue quality and a short delay to surgery [8,9,10,11]. One known surgical technique is arthroscopic ACL repair using an augmentation technique, as augmentation has been shown to have a crucial role in mechanical protection of the ACL, allowing for a self-healing response and the formation of stable scar tissue [11,12,13]. The first clinical results from small patient cohorts have been promising and Heusdens et al. reported good to excellent functional results in 37 patients with a minimum follow-up of 24 months [11]. However, there is a paucity of studies investigating this approach in larger patient cohorts. Furthermore, previous studies have preferably used this technique in isolated ACL tears, while in everyday practice, typical concomitant ligamentous, meniscal and chondral injuries are present. Thus, the purpose of this study was to report if primary arthroscopic ACL repair using suture augmentation (SA) would result in good functional outcome scores and low revision rates in a large patient cohort with typical concomitant injuries.

## 2. Patients and Methods

**Figure 1:**
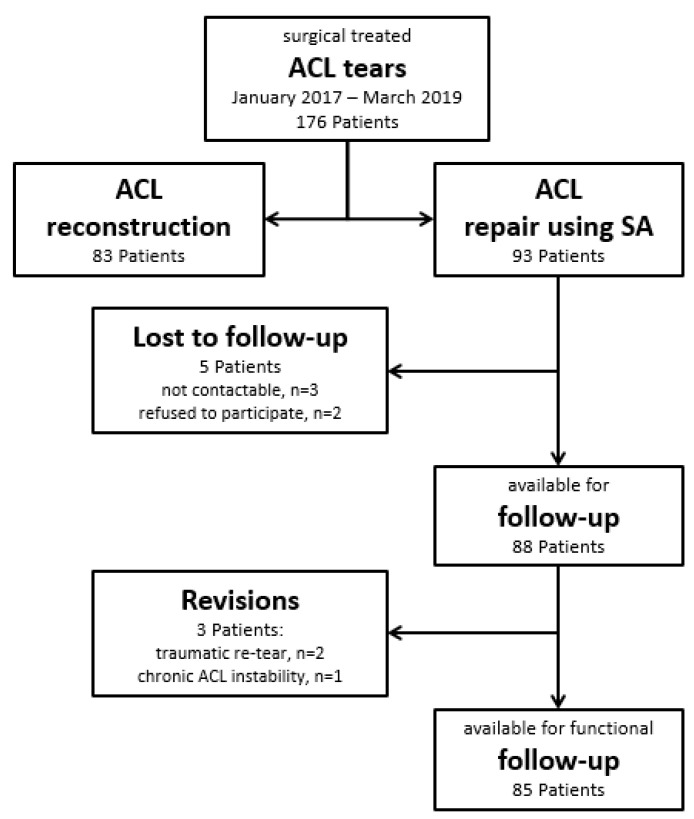
STROBE diagram of surgical treated anterior cruciate ligament (ACL) tears between January 2017 and March 2019.

This is a retrospective analysis of 93 consecutive patients (mean age 42 years, 72% female) with a minimum follow-up of 12 months who underwent primary arthroscopic ACL repair using SA between January 2017 and March 2019. All patients suffered acute ACL ruptures and underwent pre-operative magnetic resonance imaging (MRI). Patients with pre- or intraoperative mid-substance or a distal ACL tear pattern and/or poor tissue quality of the ACL remnant were not considered candidates for repair, but underwent ACL reconstruction with tendon autografts (Figure 1) [14]. A total of 2 patients initially scheduled for an ACL repair were intraoperatively converted to an ACL reconstruction due to insufficient tissue quality of the ACL remnant. No patient initially scheduled for an ACL reconstruction was intraoperatively eligible for an ACL repair. Reasons for injury were skiing (91%), snowboard (3%), free-ski (2%), sledding (1%), soccer (1%) and mountain biking (1%). All surgical repairs were preferably performed on the day of injury and the reasons for delayed surgery included: need for insurance clearance, patient’s expressed wish or because the patient was received as an external referral. The senior author performed all surgeries but was not involved in data collection and analysis.

### 2.1. Surgical Technique and Rehabilitation

**Figure 2:**
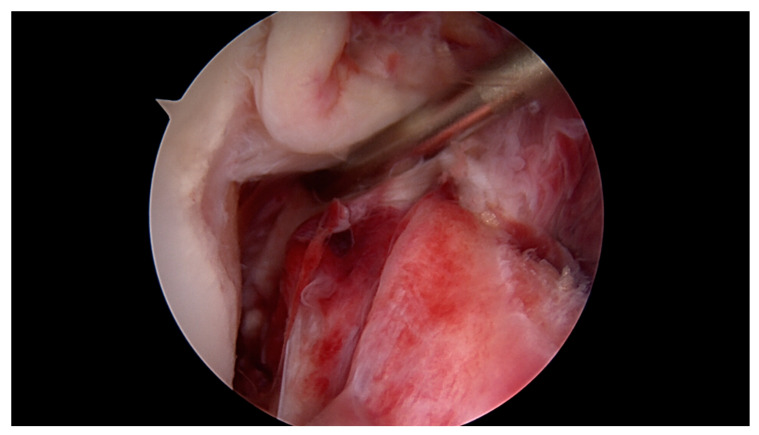
Proximal ACL tear 5 h after injury with excellent tissue quality and intact synovial coverage of the ACL remnant.

**Figure 3:**
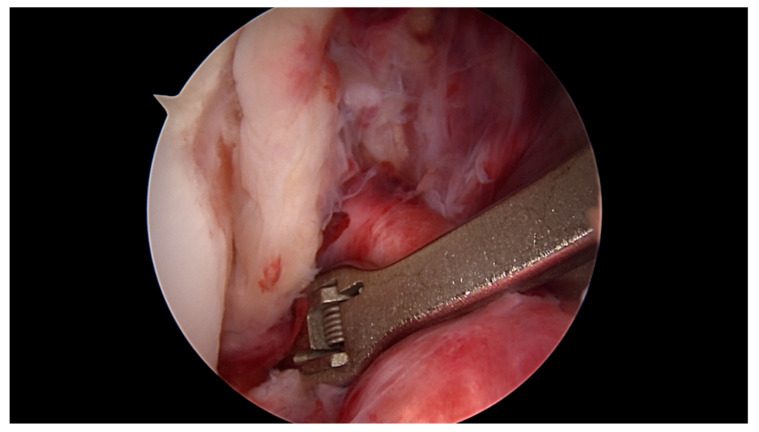
The labral scorpion suture passer is placed approximately 1 cm distal to the ACL tear.

**Figure 4:**
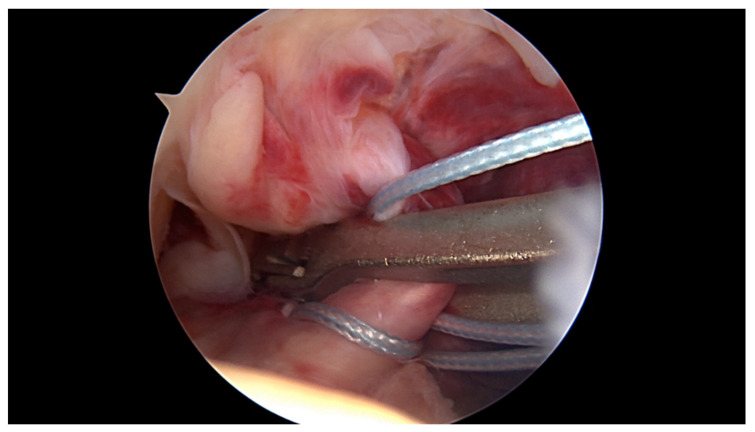
Using the labral scorpion suture passer, a No. 2 FiberWire^®^ is passed 3 times through the ACL remnant.

**Figure 5:**
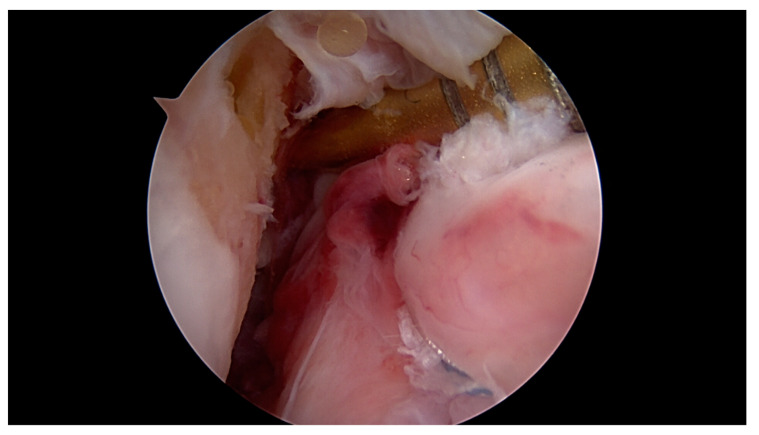
An arthroscopic 45 degree awl is used to perform focused microfracture near the femoral.

**Figure 6:**
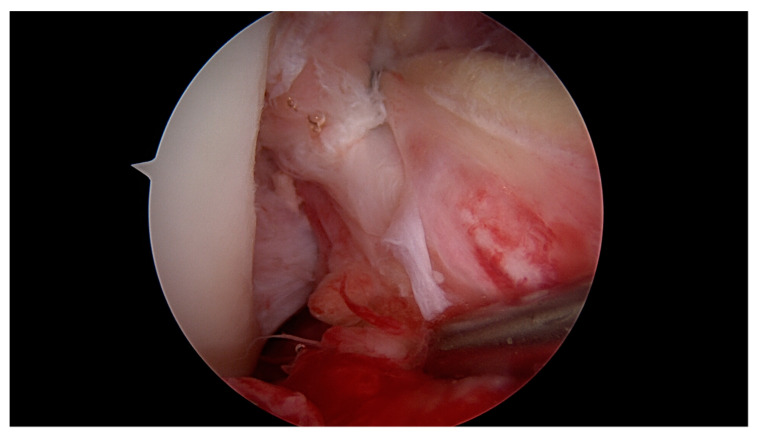
Completed arthroscopic ACL repair using suture augmentation (SA).

All surgeries were performed under spinal anaesthesia with the affected leg placed in an adjustable leg holder and with administration of an intravenous antibiotic prophylaxis. An examination under anaesthesia of the knee was performed prior to surgery in order to confirm ACL instability. The standard anterolateral and anteromedial portals were established to perform the arthroscopy to confirm all suspected findings with a probe. The ACL was assessed regarding tear pattern and tissue quality. When a proximal tear pattern with good tissue quality was identified (Figure 2), the patient was deemed eligible for arthroscopic ACL repair using SA: a labral scorpion suture passer (Arthrex, Naples, FL, USA) was used to pass a No. 2 FiberWire^®^ (Arthrex, Naples, FL, USA) 3 times approximately 1 cm distal to the tear through the ACL remnant (Figure 3 and Figure 4). The femoral tunnel was drilled in anatomic manner within the footprint with 130 degrees knee flexion using a spade tip drill pin (Arthrex, Naples, FL, USA). An arthroscopic 45 degrees awl was used to perform a microfracture near the femoral footprint to enhance healing (Figure 5). Afterwards, a shuttling loop was passed through the femoral tunnel. The tibial drilling guide (Smith and Nephew, Andover, MA, USA) was placed at the anterior centre of the tibial footprint and a small skin incision at the anteromedial aspect of the proximal tibia was established to allow drilling and shuttling of a loop through the tibial tunnel. The tibial and femoral shuttling loop as well as the FiberWire^®^ suture were retrieved through the anteromedial portal, the latter two placed in the tibial shuttling loop and passed through the tibial tunnel. Using the femoral shuttling loop, the SA construct containing a FiberTape^®^ (Arthrex, Naples, FL, USA) armed TightRope^®^ (Arthrex, Naples, FL, USA) and the FiberWire^®^ were carefully shuttled through tibia and femur so that the TightRope^®^ button flipped at the femoral cortex. The FiberTape^®^ was fixed distally at the anteromedial tibia with a 4.75 SwiveLock^®^ (Arthrex, Naples, FL, USA) with the knee in full extension. Subsequently, the FiberWire^®^ suture and TightRope^®^ tensioning suture were knotted on tension completing the ACL repair (Figure 6). Concomitant meniscal injuries were addressed either with direct repair (RapidLoc^®^, Mitek Products, Westwood, MA, USA) or with cautious partial resection when repair was deemed not feasible due to the configuration of the tear or pre-existing degenerative meniscopathy (Table 1). Concomitant chondral injuries were carefully smoothed or prudently underwent nano-fracture under direct vision.

Postoperatively, all patients were administered with a standardized ACL rehabilitation protocol with short-term immobilization in a full extension neoprene knee splint until sufficient leg stabilization was regained (after 1–2 days). In full extension, full weight bearing on crutches (for 6 weeks) was allowed until the pain threshold. Passive mobilization of the knee started immediately on the first post-operative day, followed by increasing active and assistive mobilization, including mobilization of the patella. Knee flexion was limited to 90° for 4 weeks. No quadriceps activities against distal resistance or in open-chain exercises were allowed for 3 months. Patients who underwent meniscal repair were administered to partial weight bearing and a maximum of 90° knee flexion for 4 weeks. Physiotherapy (2–3 x/week) and aquatic therapy (1 x/week) completed the postoperative care. Patients were followed up 1 week, 4 weeks, 3 months, 6 months, 12 months, 18 months and 24 months postoperatively with critical assessment of treatment goals.

### 2.2. Follow-Up Examinations

Patients’ reported outcome measures were determined at latest follow-up using standardized scoring systems: International Knee Documentation Committee Score (IKDC) Subjective Knee Evaluation Form, Lysholm score (LS) and Tegner score (TS). Clinical assessment included assessing knee laxity using the KT-1000 arthrometer (Med Metrics Corp. Inc., San Diego, CA, USA) with a 134N load in comparison to the uninjured side [15].

### 2.3. Statistical Analysis and Clinical Relevance

Statistical analysis was performed using Excel 12.3.6 (Microsoft Corp., Redmond, Washington, USA) and SPSS 25.0 (IBM Corp., Armonk. New York, NY, USA). Frequencies were calculated for categorical variables and normality testing was performed using the Kolmogorov–Smirnov test. Depending on the distribution of data, metric variables were compared using the Mann–Whitney U test for non-parametric analysis and Student’s t-test for parametric analysis. For non-parametric data, the Hodges–Lehmann estimator was used to estimate the median of differences with 95% confidence intervals (CI). Statistical significance was set at 0.05 and all *p*-values reported are two-sided. Clinical relevance was evaluated using the minimal detectable change (MDC) with its reported values for the IKDC (8.8–15.6), LS (8.9) and TS (1) [16,17].

The study was approved by our local ethics committee (No. 2019-00758) and performed in accordance with the Declaration of Helsinki.

## 3. Results

Eighty-eight patients (67 female) with a mean BMI of 24 (range 18–35) and a mean age on the day of surgery of 42 years ± 13 were available with a mean follow-up of 21 months (range 12–39, follow-up rate: 95%). The mean delay from injury to surgery was 1.5 days (range 0–18) and 37 patients (44%) were treated on the day of injury.

Several concomitant injuries were observed and addressed intraoperatively: of the 27 vertical lateral meniscus lesions, 15 underwent partial resection (56%), 6 had a primary repair (22%), 2 bucket handle lesions were resected (7%) and 4 were repaired (15%), respectively. Of the 29 medial meniscus lesions, 15 were resected (52%) and 4 were repaired (14%), while 5 bucket handle lesions were resected (17%) and 5 were repaired (17%). Regarding the 49 medial collateral ligament lesions, 19 strains (39%), 9 partial lesions (18%) and 21 total ruptures without avulsion fragments (43%) were observed. For the 27 lateral concomitant ligamentous lesions, we observed 23 strains of the lateral collateral ligament (85%) and 4 sustained Segond fractures of the anterolateral ligament (15%). All collateral ligament lesions were stable during clinical examination under anaesthesia and required no further surgical treatment.

Patients who required revision surgery were excluded for analysis of functional results. In the remaining 85 patients, mean IKDC score was 87.4 ± 11, mean LS was 92.6 ± 11, mean pre-traumatic TS (TS_Pre_) was 6 ± 2 and mean postoperative TS (TS_Post_) was 6 ± 2 with a mean difference (TS_Diff_) of 1 ± 1. KT-1000 measurements were available in 34 patients and showed a mean side-to-side laxity of 1 mm (range 0–4).

Concomitant injuries had no significant impact on postoperative functional outcome scores (Table 1). Likewise, same-day surgery versus a delay had no significant impact on IKDC (*p* = 0.228), LS (*p* = 0.377) and TS_Diff_ (*p* = 0.572). No significant differences were observed, when patients were grouped according to gender, age, BMI or delay in surgery (Table 2). Furthermore, the estimated medians of differences for IKDC, LS and TS_Diff_ did not exceed the MDC for the respective scores (Table 1 and Table 2).

Clinically meaningful differences were included in the 95% CI of the IKDC for concomitant injuries, posterolateral fractures and BMI > 30, in LS for concomitant posterolateral fractures and BMI > 30 and in TS_Diff_ for concomitant posterolateral fractures, lateral collateral ligament injuries and BMI >30. When assessing for true existence according to Harris et al., the differences in IKDC (for concomitant injuries and BMI > 30), in LS (for BMI > 30) and in TS_Diff_ (for concomitant posterolateral fractures and for lateral collateral ligament injuries) represent true negative findings, as they have demonstrated no statistical significance and the upper limit of the respective 95% CI is less than the MDC. For the observed differences in IKDC and in LS for concomitant posterolateral fractures as well as in TS_Diff_ for BMI > 30, the outcome is inconclusive as it is not statistically significant, but the 95% CI overlaps the MDC [18].

A total of three patients (3%) underwent revision surgery: one patient complained of persistent ACL instability (KT-1000 side-to-side laxity of 9 mm) and underwent ACL reconstruction 3 months postoperatively. Two patients suffered a traumatic ACL re-tear (12 and 15 months postoperatively, respectively) that were treated with a same-day secondary ACL repair in the first patient and a complication-free ACL reconstruction in the latter patient. While the first patient experienced a severe ski fall with twisting of the left knee and an immediate audible popping noise, the second patient was fouled playing soccer [19]. No further surgeries (e.g., for subsequent meniscal or chondral lesions) were reported.

## 4. Discussion

The main findings of this study are: (1) revision rates in arthroscopic ACL repair using SA remain low. (2) Typical concomitant injuries do not negatively affect the postoperative functional outcome and (3) same-day surgery versus a short-term delay of up to 18 days has no statistically significant or clinically relevant impact on patient-reported postoperative functional outcome scores. (4) Overall, arthroscopic ACL repair using SA provides good to excellent patient-reported short-term functional outcome in a large patient cohort.

The reported revision rate in this cohort is lower than those of previously reported publications, which range between 4.8% and 9% [11,12,13]. These might be attributed to small sample sizes [8] or lower patients’ age [11,12], as Meister et al. and Henle et al. showed for dynamic intraligamentary stabilization (DIS) that younger patients tend to be more prone to a re-tear [20,21]. However, we were not able to identify age or pre-traumatic level of activity as a risk factor for a re-tear. Thus, other reasons such as patient selection or possible technical errors linked to a learning curve with a new surgical technique might be causative but remain speculative [22].

Concomitant chondral, meniscal and ligamentous injuries are frequently observed in patients suffering an ACL tear [23,24]. Especially for concomitant meniscal pathologies, varying functional results are reported in ACL reconstruction [25,26,27]. Considering that we did not find differences in functional outcome in patients with concomitant meniscus injuries, given that we addressed all meniscal injuries encountered in the same surgery, ACL repair appears to be a good option if meniscus injuries are present and can be addressed in the same surgery. In line with our results, Jonkergouw et al. reported good objective and patient-reported outcome parameters following ACL repair with and without additional suture augmentation in patients with acute proximal ACL injuries and concomitant medial and lateral meniscal injuries [13]. Furthermore, DiFelice et al. argued for a delayed surgery when a concomitant MCL injury was present [8]. While we agree that severe ligamentous injuries of the collateral ligaments might lead to a high risk of subsequent instability and potential failure of the repair, we were not able to detect significant statistical or truly clinically relevant differences in postoperative patient-reported functional outcome scores or an increased risk for revision surgery in patients suffering concomitant ligamentous injuries in our cohort [18].

Critical patient selection is essential in arthroscopic ACL repair, with tear pattern, tissue quality and time to surgery being reported to be key factors [8,28,29]. However, a certain delay in surgery often remains unavoidable in everyday practice as DiFelice et al. report a delay of on average 39 days and other authors only stating they performed surgery “within 3 months of initial injury” [8,11,12]. Magarian et al. showed that a delay of 2 or 6 weeks adversely affects the functional outcome in augmented primary repair of porcine ACL, and Murray et al. demonstrated the strength of time-dependent histological response after ACL rupture with retraction and lack of healing [30,31]. Despite the fact that 37 patients were treated on the day of injury, we are not able to report differences compared to patients with a short delay in surgery (of up to 18 days). This is possibly due to the fact that all patients were operated on very early compared to the recommendations of previous studies [8,10]. Given the histological findings, we still recommend performing ACL repair as early as possible; however, considering the excellent functional results reported in the present study as well as in the aforementioned studies, a delay of several weeks up to 3 months does not appear to be clinically relevant with the numbers available.

Functional outcome scores in our cohort are similar to those previously reported by other authors: Jonkergouw et al. reported an IKDC of 89, LS of 93, TS_Pre_ of 7.0 and TS_Post_ of 6.4 in 25 patients with a mean follow-up of 2.4 years [13] and Heusdens et al. a KOOS Pain of 87.9, KOOS Symptoms of 84.5, KOOS ADL of 93.3, KOOS Sport of 76.6 and a KOOS QOL of 70.8 in 37 patients at 24 months follow-up [11]. However, the literature is lacking functional outcome results of large patient cohorts.

The arthroscopic ACL repair technique with the most experimental and clinical data available is DIS [32,33,34,35,36]. Although several authors reported functional results comparable to our cohort, Ahmad et al. reported low DIS survival rates (of only 70% at 5-year follow-up) and Osti et al. reported a high rate of recurrent instability (of up to 17.5%) [22,32,35,37,38]. While Ahmad et al. concluded that they were lacking evidence of the ideal surgical candidate during patient enrolment and stricter indications would have likely resulted in an overall better outcome, first randomized clinical trials show that functional results and clinical failure of DIS are comparable to ACL reconstruction [37,38]. Although the understanding of the ideal surgical candidate for ACL repair has evolved over the past few years and critical patient selection was routinely performed in the reported cohorts, no randomized controlled trial comparing SA and ACL reconstruction has yet been performed [10,35].

We acknowledge several limitations to our study. (1) Nearly all patients suffered an ACL tear due to winter sport injuries, excluding typical ACL injury patterns such as soccer or football. Weaver et al. reported a high incidence of a proximal ACL tear pattern in skiing accidents, a tear pattern that is crucial for arthroscopic ACL repair [10,39]. Thus, it remains unclear whether these results can be reproduced for injuries due to non-winter sports injuries. (2) The mean age of the patients treated in our study is high, also in comparison to other studies on ACL repair using SA [12,13,40]. This is possibly due to the higher patient age in the ski region where this study was conducted. However, the Tegner score reported preoperatively is high and comparable to other studies and we included several professional athletes in our study. (3) While we were not able to identify a difference in knee laxity using the KT-1000 arthrometer, we must acknowledge that these measurements were not available for all patients. However, several previous studies have demonstrated good knee stability following ACL repair using SA and only 2 of our 34 measured patients had a side-to-side difference of >3 mm [11,13,41]. As the knee stability and at 12- or 18-month follow-up can simply reflect the strength of the SA itself, the long-term follow-up of our cohort will show whether ACL healing is sufficient or whether knee instability occurs with potential long-term wear/tears of the FiberWire^®^. (4) While we were unable to detect truly meaningful clinical relevant differences in patient-reported outcome parameters based on the MDC, an additional assessment of the minimal clinically important difference (MCID) would have allowed for a more distinct pre- versus postoperative comparison. However, due to our study design, preoperative patient-reported outcome parameters that are required to determine the MCID are not available and the analysis is thus beyond the scope of this manuscript.

## 5. Conclusions

Primary ACL repair using SA provides good to excellent postoperative functional outcome in a large patient cohort with a minimum follow-up of 12 months. Patient’s age (>40 years), BMI (>30) and coexisting ligament or meniscal injuries did not seem to influence postoperative functional results and revision rates remain lower than previously reported.

## Figures and Tables

**Table 1:** Concomitant injuries and their respective impact on the median postoperative functional outcome scores of the injured/non-injured patients.

		Concomitant Injury Present		
		Yes vs. No (Estimated Median; 95% CI)		
	Patients (%)	IKDC *	LS *	TS_Diff_ *
Concomitant injuries	76 (86%)	90.8 vs. 92.0	95 vs. 95	0 vs. 0
		(−3.4; −8, 2.3)	(0; −5, 1)	(0; 0, 0)
		*p* = 0.239	*p =* 0.384	*p =* 0.858
Posterolateral fracture	10 (11%)	85.7 vs. 90.8	95 vs. 95	0 vs. 0
		(4.6; −2.3, 12)	(5; 0, 11)	(0; −1, 0)
		*p =* 0.226	*p =* 0.108	*p* = 0.103
Meniscus	49 (56%)	90.8 vs. 89.7	95 vs. 95	0 vs. 0
		(0; −4.6, 2.3)	(0; −3, 2)	(0; 0, 0)
		*p =* 0.741	*p =* 0.946	*p =* 0.968
medial	29 (33%)	90.8 vs. 90.3	95 vs. 95	0 vs. 0
		(−2.3; −5.7, 1.2)	(0; −4, 2)	(0; 0, 0)
		*p =* 0.22	*p* = 0.731	*p =* 0.419
lateral	27 (31%)	90.8 vs. 89.7	95 vs. 95	0 vs. 0
		(0; −4.6, 3.5)	(0; −3, 4)	(0; 0, 0)
		*p =* 0.925	*p* = 0.882	*p* = 0.953
Collateral ligament	54 (61%)	88.5 vs. 93.1	95 vs. 95	0 vs. 0
		(3.5; 0, 6.9)	(0; −1, 4)	(0; 0, 0)
		*p =* 0.053	*p =* 0.578	*p =* 0.95
medial	49 (56%)	88.5 vs. 92.6	95 vs. 95	0 vs. 0
		(2.3; −1.1, 5.8)	(0; −4, 1)	(0; 0, 0)
		*p =* 0.144	*p =* 0.889	*p =* 0.782
Lateral ^†^	27 (31%)	89.7 vs. 90.8	95 vs. 95	1 vs. 0
		(2.3; −1.2, 5.8)	(1; −1, 5)	(0; −1, 0)
		*p* = 0.256	*P* = 0.285	*p* = 0.107

^†^ including 3 Segond fractures of the anterolateral ligament. * Three patients who underwent revision surgery were excluded. Estimated medians of differences and 95% CI were calculated with the Hodges–Lehmann estimator. *p*-value was calculated with the Mann–Whitney U test.

**Table 2:** No significant impact of gender, age, BMI and delay in surgery on median postoperative functional outcome scores. * Three patients who underwent revision surgery were excluded. Estimated medians of differences and 95% CI were calculated with the Hodges–Lehmann estimator. *p*-value was calculated with the Mann–Whitney U test.

		Yes vs. No (Estimated Median; 95% CI)		
	Number of Patients	IKDC *	LS *	TS_Diff_ *
Male vs. Female	24 vs. 61	91.4 vs. 89.7	99.5 vs. 95	0 vs. 0
		(2.3; −1.2, 5.7)	(2; 0, 5)	(0; 0, 0)
		*p* = 0.304	*p* = 0.061	*p* = 0.943
Age > 40 years vs. ≤40 years	56 vs. 29	90.8 vs. 90.8	95 vs. 95	0 vs. 0
		(−1.1; −4.6, 2.3)	(0; −4, 2)	(0, 0, 0)
		*p* = 0.512	*p* = 0.989	*p* = 0.651
BMI > 30 vs. ≤30	6 vs. 79	86.8 vs. 90.8	95 vs. 95	0.5 vs. 0
		(2.3; −3.4, 8)	(0; −5, 5)	(0; −1, 1)
		*p* = 0.344	*p* = 0.892	*p* = 0.88
Same-day surgery vs. Delay	37 vs. 48	90.8 vs. 90.8	95 vs. 95	0 vs. 0
		(1.2; −2.3, 4.6)	(0; −4, 1)	(0; 0, 0)
		*p* = 0.47	*p* = 0.957	*p* = 0.968
